# Blarcamesine in Early Alzheimer Disease Phase 2b/3 Randomized Clinical Trial

**DOI:** 10.1002/alz.090729

**Published:** 2025-01-09

**Authors:** Marwan N. Sabbagh, William R. Chezem, Kun Jin, Christopher U Missling, Anavex Life Sciences Research Group

**Affiliations:** ^1^ Barrow Neurological Institute, Phoenix, AZ USA; ^2^ Anavex Life Sciences Corp, New York, NY USA; ^3^ Anavex Life Sciences, New York, NY USA; ^4^ Anavex Life Sciences Corp., New York, NY USA

## Abstract

**Background:**

There are no approved oral disease‐modifying treatments for Alzheimer disease (AD). This study was intended to assess efficacy and safety of blarcamesine (ANAVEX^®^2‐73), an orally available small‐molecule activator of the sigma‐1 receptor (SIGMAR1) designed to exert neuroprotection through restoration of cellular homeostasis including autophagy enhancement.

**Method:**

ANAVEX^®^2‐73‐AD‐004 is a multicenter (52 medical research centers/hospitals in 5 countries), randomized, double‐blind, placebo‐controlled, 48‐week phase 2b/3 trial that enrolled 508 participants with early AD (mild cognitive impairment/mild dementia) from July 2018 to June 2021 (last patient visit in June 2022). Participants were randomized to receive blarcamesine (n = 338) or placebo (n = 170) oral capsules once daily for 48 weeks.

The co‐primary cognitive and functional outcomes were the change in ADAS‐Cog13 and ADCS‐ADL scores from baseline to 48 weeks. Additional clinical outcomes include the secondary outcome CDR‐SB and the exploratory outcome CGI‐I. All clinical endpoints were analyzed using a mixed model for repeated measures (MMRM).

**Result:**

The blarcamesine group demonstrated improvement compared to the placebo group in all clinical endpoints at 48 weeks: when comparing least‐squares mean (LSM) change in ADAS‐Cog13 score (difference, −1.783 [95% CI, −3.314 to −0.251]; P = 0.0226), LSM change in ADCS‐ADL score (difference, 1.019 [95% CI, −0.66 to 2.70]; P = 0.234), LSM change in CDR‐SB score (difference, −0.456 [95% CI, −0.831 to −0.080]; P = 0.0175), and LSM change in CGI‐I score (difference, −0.285 [95% CI, −0.474 to −0.095]; P = 0.0033). Participants with ≥1 serious treatment‐emergent adverse events (TEAEs) occurred in 56 participants (16.7%) in the blarcamesine group and 17 (10.1%) in the placebo group. Common treatment‐emergent adverse events included dizziness, which was mostly mild to moderate in severity.

**Conclusion:**

In this phase 2b/3 randomized clinical trial, we found that among participants with early AD, blarcamesine significantly slowed clinical progression on prespecified primary outcome global and cognitive measures at 48 weeks in the ITT population. These results suggest that blarcamesine may be an effective, safe, and novel scalable oral treatment for early AD.

